# A Case Report: Gastric Mixed Neuroendocrine-Nonneuroendocrine Neoplasm with Aggressive Neuroendocrine Component

**DOI:** 10.1155/2017/9871687

**Published:** 2017-05-24

**Authors:** Quang Duy Pham, Ichiro Mori, Robert Y. Osamura

**Affiliations:** ^1^Center for Diagnostic Pathology, Mita Hospital, International University of Health and Welfare, Tokyo 108-8329, Japan; ^2^Department of Pathology, Cho Ray Hospital, Ho Chi Minh City, Vietnam; ^3^Women's Oncology Center, Sanno Medical Center, International University of Health and Welfare, Tokyo 108-8329, Japan

## Abstract

Mixed neuroendocrine-nonneuroendocrine neoplasm (MiNEN) is defined as mixed epithelial neoplasms composed of both neuroendocrine and nonneuroendocrine components with variable proportions for each component. Neuroendocrine component can show morphological features including well- or poorly differentiated neuroendocrine neoplasms and nonneuroendocrine component can present different tumor types depending on the site of origin. Recently, studies of tumors have shown that MiNENs are not as rare as our traditional belief, due to the wide application for immunohistochemistry. However, our knowledge of MiNENs is still limited. There is no universal consensus about nomenclature, classification, and guideline of treatment. Hereby, we would like to present a case report of gastric MiNEN with aggressive neuroendocrine component to contribute a small part towards common understanding of gastric MiNENs.

## 1. Introduction

The first description of a gastrointestinal neoplasm with mixed exocrine and neuroendocrine components was published by Cordier [1]. From that time on, a number of cases have been reported in the literature and the intensive use of immunostaining has increased the opportunities of identifying these tumors. During the last 20 years, different terms have been used as diagnostic term in pathology reports.

In the latest version of WHO Classification of Tumors of The Digestive System, these mixed neoplasms are called by the term “mixed adenoneuroendocrine carcinomas (MANECs).” Regarding WHO Classification, MANECs have both an exocrine and an endocrine component. Arbitrarily, at least 30% of either component should be identified to qualify for this definition [2]. This view has been based mainly on the assumption that a minor neoplastic component (less than 30%) is unlikely to influence the behavior. However, it is worth noting that the cut-off has been chosen arbitrarily and was not based on proven clinical evidence [3]. In fact, the MANEC does not adequately convey the morphological and biological heterogeneity of digestive mixed neoplasms and has created some misunderstanding among both pathologists and clinicians. The new term “mixed neuroendocrine-nonneuroendocrine neoplasm (MiNEN)” is being proposed to use and to replace the old terminology used by the WHO 2010 Classification.

As a rule, the presence of two morphologically recognizable components, with neuroendocrine and nonneuroendocrine features on hematoxylin and eosin stains, is required to formulate a suspect of MiNEN. The application of a correct panel of immunostaining is then mandatory to confirm the diagnosis [3]. Usually neuroendocrine markers including chromogranin, synaptophysin, and CD56 are used for recognize neuroendocrine differentiation combined with the markers on nonendocrine differentiation such as CDX2 and CEA.

MiNENs have been described in several organs and the development of immunohistological methods has contributed greatly to the recognition in daily diagnostic practice. However, after all, these tumors are rare, notably in stomach. To date, in English literature, only seven cases were reported to have occurred in the cecum and less than 40 cases in the stomach [4].

As the lack of complete understanding about pathogenesis of MiNENs, appropriate treatment for patients has not proved yet. The prognosis for gastric MiNENs is quite poor, most patients presenting with advanced-stage disease and short survival. In this paper, we present a case gastric MiNEN with neuroendocrine hepatic metastases and a brief review of the literature.

## 2. Case Description

A 68-year-old Japanese man was hospitalized with giant gastric tumor identified by endoscopy. He had not had a significant symptom, except for discomfort in abdomen one year ago. He had smoked 40 tobaccos a day for 10 years and drunk 5 glasses of whisky a day for 5 days/week. Moreover, he had prostate hyperplasia and hypertension in his medical history. No remarkable information was recorded in family medical history.

**Figure 1:**
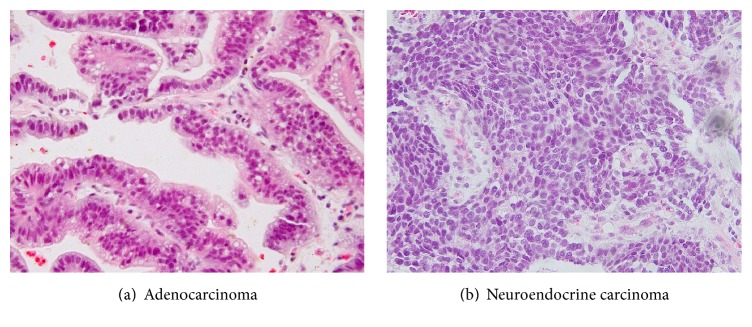
Microscopy of gastric tumor on biopsy.

**Figure 2:**
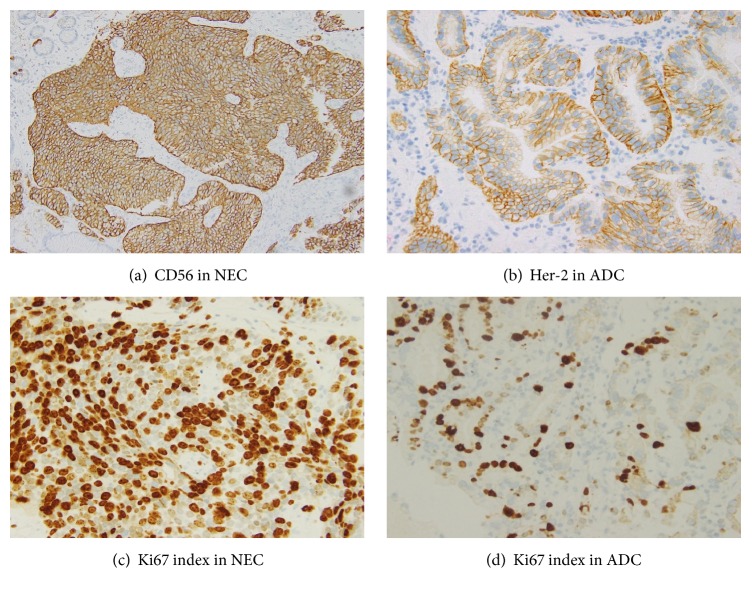
Immunoprofile of gastric tumor performed on biopsy.

Since fecal occult blood detected by routine health check-up, he underwent biopsy of stomach by endoscopy and specimen was sent to Department of Pathology. Microscopically, submitted specimen was composed of malignant epithelial and neuroendocrine components. The epithelial component was mainly composed of tall columnar cells with a distinct tubular structure. The nuclei are enlarged, hyperchromatic, and pleomorphic with loss of nuclear polarity. The malignant epithelial cells have eosinophilic cytoplasm with abundant mucus production. They were classified into well-differentiated and moderately differentiated tubular adenocarcinoma (tub1 and tub2) based on JGCA 2010 Classification of Gastric Carcinoma (6) (Figure 1). The neuroendocrine component has nesting and trabeculae patterns. Tumor cells have round, ovoid, or spindled nuclei and scant cytoplasm. Cell borders are rarely seen. Nuclear chromatin is finely granular and nucleoli are absent or inconspicuous. Based on nuclear structure and cell size of neuroendocrine component, the diagnosis was small cell carcinoma corresponding WHO 2010 Classification of Tumors of the Digestive System (Figure 1). Therefore, the pathological diagnosis was considered as gastric mixed adenoneuroendocrine carcinomas (MANEC) as stated in WHO 2010 Classification. It was then confirmed by immunostaining with neuroendocrine component positive for synaptophysin (3+), chromogranin A (3+), and CD56 (2+) (Table 2; Figure 2). We also verified expression of HER2 for targeted therapy and the proliferative index by Ki67. The result was HER2 (3+) only in adenocarcinoma component and Ki67 labelling index is strongly positive (Table 2; Figure 2). By scrutinised cT4a (SE) N3b M1, gastric cancer was diagnosed at stage IV. At the same time, hepatic metastases were also found.

Laboratory test revealed tumor markers (Table 1).

After the first pathological report as poorly differentiated adenocarcinoma, clinicians decided to start chemotherapy (S-1, CDDP) as soon as possible due to advanced condition of our patient. The additional report of immunohistochemistry confirmed the presence of neuroendocrine component and the adenocarcinoma component was strongly positive for HER2. Therefore, clinicians added Trastuzumab in chemotherapy regime to control tumor progression.

Our patient was given total 5 cycles of chemotherapy (S-1, CDDP, Trastuzumab) but liver lesions tended to increase markedly by interval CT-scan checking and hence main treatment shifted to neuroendocrine carcinoma (NEC) with Sandostatin-LAR. Before starting therapy with somatostatin analogue, we carefully checked expression of somatostatin receptors and mTOR peptide by immunostaining. His result was SSTR2 (2+), SSTR5 (2+), and mTOR (−). Totally, this patient was given 2 injections. Eventually, he was given palliative treatment without surgery due to his general weakness. He died as a result of liver failure after 7 months treatment in total.

The hospital autopsy was performed to help answer specific questions about the cause of death. His body was carefully examined and we found many lesions at different organs. On macroscopic examination, the primary tumor was on middle part and the lesser curvature of stomach (M less). The macroscopic type was type 3 which means infiltrative and ulcerative type [5]. The tumor was measured 9 × 6 cm in size. Metastatic lesions could be found in liver, diaphragm, pancreas, and periaortic lymph nodes as multinodular appearance and all of them are comprised of only neuroendocrine carcinoma component.

**Figure 3:**
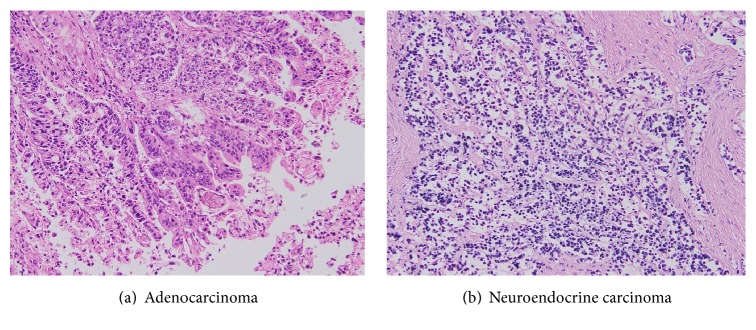
Microscopy of gastric tumor on autopsy.

**Figure 4:**
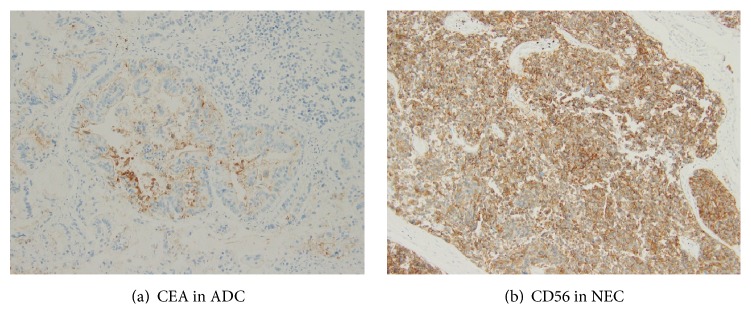
Immunoprofile of gastric tumor performed on autopsy.

**Figure 5:**
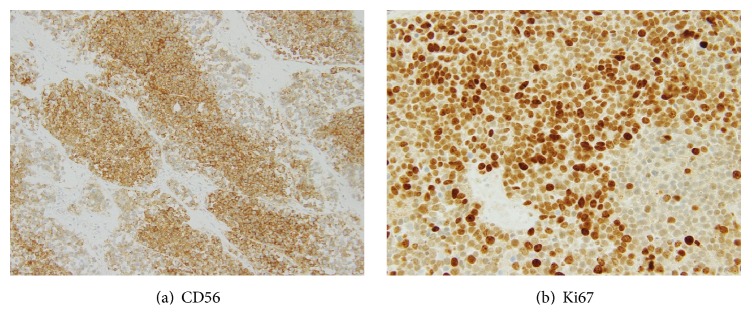
Immunoprofile of liver lesion performed on autopsy.

By careful autopsy examination, the final conclusion was gastric MANECs with neuroendocrine carcinoma component metastasis to liver, pancreas, diaphragm, and periaortic lymph nodes. Adenocarcinoma was not detected in the metastatic lesions and liver failure, due to metastasis of NEC, was the cause of death (Figures 3, 4, and 5).

To confirm our diagnosis of MANEC as definition in WHO 2010 Classification, we ordered for immunostaining on autopsy slides and summarized immunoprofile as in Table 2. In general, adenocarcinoma component took place 10–20% areas of the gastric tumor, which was confirmed by autopsy examination.

## 3. Discussion

The term MANEC was used to define the category of mixed neoplasm in the 2010 WHO Classification of Tumors of Digestive system. However, it has created some misunderstanding and has been a matter of debate among pathologists and clinicians as it seems to imply that all MANECs are composed of adenocarcinoma and NEC, so they have to be managed with the specific treatments approved for these specific tumor types [3]. Indeed, the spectrum of these tumors encompasses all the possible combination between neuroendocrine neoplasms (NET and NEC) and other epithelial tumors of tubular digestive tract (adenomas, adenocarcinoma, and squamous cell carcinoma) [3].

NECs account for 6–16% of gastric neuroendocrine neoplasms and no specific epidemiological data are available for MANECs according to the 2010 WHO Classification. In Japan, a nationwide survey analysis of epidemiological trends of pancreatic and gastrointestinal neuroendocrine tumors was established in 2010. An estimated 8,088 people received treatment for GI-NETs (gastrointestinal neuroendocrine tumors) in Japan in 2010, which means the prevalence of the patients with GI-NETs was about 6.42 per 100,000 people. The frequency of NEC among all GI-NETs was 6.2%. NEC was most common among foregut NETs (12.6%) followed by midgut NETs (9.1%) and hindgut NETs (2.3%). Epidemiological data for gastric MiNENs in Japan have not been determined yet [6]. To date, mostly data on gastric MiNENs are from case reports. For this reason, standard therapy is still lacking for gastric MiNENs and there seemed to be no improvement in outcomes.

The average age of patients is 59.3 ± 10.9 (the age at onset of our patient was 67) and male-to-female ratio was 2.4 : 1 or higher [7, 8]. Gastric NEC and MiNEN usually present with clinical nonspecific symptoms similar to those of conventional gastric cancer, often at an advanced stage, with distant metastases [6, 9, 10]. For this reason, majority of gastric MiNENs present with an aggressive behavior and poor prognosis. The median survival time for MiNENs is less than 12 months [11, 12]. Our patient died of this disease with the treatment length of 7 months in total. It seems to be shorter than in other studies as nonsurgical treatment due to his general condition.

According to the 2010 WHO Classification, it is likely that NECs and MANECs share the complex pathogenetic setting of the common gastric adenocarcinoma [9]. These include smoking, high intakes of salt-preserved and/or smoked foods, bile reflux, and infection with* H. pylori* [13]. The available data on the genetics of NECs and MANECs are scant; however, gastric NECs, like NECs at other sites of the gastrointestinal tract, display multiple chromosomal abnormalities involving key cell-cycle regulatory genes. Data on gastric MANECs indicate a relatively higher frequency of chromosomal abnormalities in the NEC versus the adenocarcinoma component [9]. Recently, a few studies indicate that smoking and alcohol consumption were not associated with NETs (neuroendocrine tumors) development in either men or women. However, a family history of cancer and personal history of diabetes were significant risk factors for all NETs [14, 15].

About location, NECs and MANECs may arise at any site in the stomach [9]. Indeed, approximate half of these tumors were located in the upper stomach, 25% were located in the mid stomach, 20% were located in the distal stomach, and lesser than 5% were found in other sites [8, 10].

As far as we know, most of gastric neuroendocrine neoplasms (GNENs), notably mixed adenoneuroendocrine carcinomas, are highly malignant. Thus, early detection is crucial. In fact, patient with GNENs mostly presented with nonspecific symptoms [16]. Nonfunctioning tumors, as patients without clinical symptoms and with no elevation of plasma hormone levels, were 67% of total number of patients treated [6]. It really is a challenge for physicians to make diagnosis. Not only physicians but also endoscopists and pathologists tend to miss the diagnosis. Endoscopy combined with biopsy is believed to be the most sensitive method to help detect small lesions and is essential not only to confirm histopathologic diagnosis but also to localize the primary lesion [16]. However, the coincidence rate of preoperative and postoperative pathological diagnosis for primary gastric neuroendocrine neoplasms is low, which was 75.0% in grade 1, 72.7% in MANEC, and 25.0% in grade 3, respectively. Therefore, it should be very cautious when diagnosis of this disease is made in a preoperative biopsy [8, 17].

Indeed, the definition for MANECs as in 2010 WHO Classification has caused difficulty in daily practice. As we known, biopsy specimen is a tiny sample and it does not represent the whole tumor. Moreover, the cut-off 30% has been chosen arbitrarily and was not based on proven clinical evidence. That is why we think that the coincidence rate of preoperative and postoperative pathological diagnosis for gastric MANEC is low. In our case, even if the whole gastric tumor was examined by autopsy, the adenocarcinoma had just occurred in 10–20% of tumor. By the standard of the 2010 WHO Classification, we could not make an appropriate diagnosis for this patient. Therefore, we believe that the term MiNEN provides a comprehensive definition for mixed neuroendocrine-nonneuroendocrine neoplasms as recommended by other authors [3].

Apart from the regional lymph nodes, the liver was the predominant site of NET metastases [8, 11, 18]. In our case, cancer cells spread to liver and even to diaphragm, pancreas, and periaortic lymph nodes. The prognosis of gastrointestinal high-grade MiNENs largely depends on stage and type of neoplastic components, but in general, it seems to be similar to that of pure NECs suggesting that the NEC component is pivotal in determining the prognosis [3, 7, 19, 20]. Clinical performance of our patient seemed to be likely as other reports. On the other hand, Ana Maria Minaya-Bravo et al. reported a case of mixed adenoneuroendocrine carcinoma of colon with distant metastasis composed of both neuroendocrine carcinoma and adenocarcinoma [21]. Moreover, Simona Gurzu et al. also reported a case with higher aggressiveness of the exocrine component [4].

There is currently no consensus regarding treatment against gastric MiNENs. Until now, surgery has been the most important treatment for these tumors even if patients were diagnosed with advanced distant metastases. Palliative surgery plays an important role in unresected metastases and additional chemotherapy can improve the survival [6–8, 10, 11, 18, 22]. Our patient had not undergone surgery due to his general weakness. Furthermore, chemotherapy was given to our patient but it was not effective as well as expected. On the other hand, we posed a hypothesis that chemotherapy had been effective against exocrine components since no adenocarcinoma component was identified in metastatic lesions. Malignant neuroendocrine cells were so strongly positive for Ki67 in liver lesions that we believe NEC component is pivotal in determining the prognosis like the other authors.

The incidence of gastric NENs including MiNENs has been increasing in the past few decades due to development of immunological techniques. However, the understanding of gastric MiNENs is still limited owing to their rarity. The majority of patients are diagnosed at advanced stage, leading to a poor prognostic. Consequently, further researches are needed in order to clarify of these neoplasms, to determine treatment options, and hence to improve outcome of patients.

## Figures and Tables

**Table 1:** Laboratory results.

Marker	Normal range		Result		
NSE	16.3	ng/ml	**2170 **	ng/ml	High
CA 125	35.0	U/ml	**417 **	U/ml	High
CA 19-9	37.0	U/ml	2.2	U/ml	
AFP	10.0	ng/ml	**11.6 **	ng/ml	High
CEA	5.0	ng/ml	**9.7 **	ng/ml	High

**Table 2:** Summary of immunoprofile.

Marker	First biopsy	Second biopsy (interval)	Autopsy	
			Primary lesion (stomach)	Secondary lesion (liver)
Synaptophysin	NEC (3+)	NEC (3+)	NEC (2+)	NEC (3+)
CD56	NEC (3+)	NEC (3+)	NEC (3+)	NEC (3+)
Chromogranin A	NEC (2+)	NEC (2+)	NEC (2+)	NEC (3+)
CEA			ADC (2+)	
Her2	ADC (3+)	No ADC	ADC (−)	No ADC
Ki67	NEC: 50–60%		NEC: 20–30%	NEC: 30–40%
	ADC: 20–30%		ADC: 20–30%	No ADC

NEC: neuroendocrine component. ADC: adenocarcinoma component. Ki67 index is calculated by manual eye-counting in hot spot with 40x magnification.

## Abbreviation

ADC: Adenocarcinoma
NEN: Neuroendocrine neoplasm
NET: Neuroendocrine tumor
NEC: Neuroendocrine carcinoma
MANEC: Mixed adenoneuroendocrine carcinoma
MiNEN: Mixed neuroendocrine-nonneuroendocrine carcinoma.
